# The clinical spectrum of central neurocytomas ranges from benign to leptomeningeal disseminating disease: a single institutional surgical series of 33 patients

**DOI:** 10.3389/fsurg.2025.1534566

**Published:** 2025-07-18

**Authors:** A. Harun Yaşar, Ayça Erşen-Danyeli, Baran Bozkurt, M. İmre Usseli, Mustafa Güdük, Koray Özduman, M. Necmettin Pamir

**Affiliations:** ^1^ Department of Neurosurgery, School of Medicine, Acıbadem University, Istanbul, Turkiye; ^2^ Department of Pathology, School of Medicine, Acıbadem University, Istanbul, Turkiye

**Keywords:** central neurocytoma, atypical central neurocytoma, brain tumor, radiotherapy, gamma knife radiosurgery, p75NTR

## Abstract

**Background:**

Central neurocytomas (CN) are rare neuroepithelial neoplasms primarily found in the lateral ventricles. While generally considered benign, their clinical behavior varies, with some cases displaying atypical features associated with increased recurrence risk.

**Material and methods:**

This is a retrospective analysis of 33 adult CN patients that were operated and followed over a 25-year period by a single surgeon. Demographic (age, gender), anatomical (localization), histopathological (atypical histology, Ki67 index, p75NTR expression), extent of resection (GTR vs. STR), adjuvant treatments (radiotherapy and radiosurgery) were analyzed as potential prognostic factors.

**Results:**

Gross total resection (GTR) was associated with favorable outcomes, with adjuvant radiotherapy effective after subtotal resection (STR). Notably, a subset of aCN cases exhibited p75NTR immunopositivity, suggesting its potential as a prognostic marker for aggressive behavior. Leptomeningeal dissemination was rare but observed in one case.

**Conclusion:**

CN cases demonstrate clinical heterogeneity, emphasizing the importance of tailored management. Close follow-up is crucial, particularly in atypical cases, to optimize patient outcomes. GTR remains the goal in surgery, while p75NTR expression may serve as a prognostic indicator but further research is needed to validate p75NTR as a prognostic factor in CN.

## Introduction

In 1982, Hassoun et al. identified a distinct group of tumors which showed neuronal differentiation without malignant features such as cellular atypia, increased mitotic activity or necrosis, and called them “central neurocytomas (CN)” (1). These rare neuroepithelial tumors arise in the lateral ventricles near the foramen of Monro and account approximately 0.1%–0.5% of all brain tumors (2–6). They usually affect young adults and show no gender predilection (7, 8). Although the exact cell of origin remains uncertain, subependymal or septal progenitor cells are the most likely source (9).

Although CNs are considered World Health Organization (WHO) grade 2 tumors with slow growth and neuronal differentiation, a subtype -atypical central neurocytoma (aCN)- exhibits atypical features and increased recurrence risk (10–14). However, aCNs have not yet been officially assigned a higher grade in the WHO 2021 classification (2).

Patients with CN typically present with increased intracranial pressure symptoms related to obstructive hydrocephalus. As a result, surgical resection remains the cornerstone of treatment. Surgical treatment, particularly when a gross total resection (GTR) is achieved, has shown excellent outcomes, with a 10-year overall survival (OS) rate reaching 90% and progression-free survival (PFS) rate of 72% (8, 15). The 5-year OS and PFS rates were 92% with GTR, while the rates were 72% and 60% with subtotal resection (STR), respectively (16). Adjuvant treatment modalities, such as fractionated radiotherapy (RT), stereotactic radiosurgery or even chemotherapy may be used (17–22).

When stratified by histopathological subtype, aCN consistently demonstrates worse outcomes. Reported 5-year OS and PFS rates are 70% and 46% for aCN, compared to 95% and 81% for typical CN, respectively (23).

Evidently, the clinical presentation and course of CN is quite variable. This study aims at analyzing the clinical features of a large, single institutional cohort to provide evidence on variability of central neurocytoma tumor biology.

## Materials and methods

### Study design

This is a retrospective analysis of a patient cohort with CN that has been operated by the senior author (MNP) over a 25-year period (January 1997 to September 2022). All patient records for this duration were reviewed and all adult patients with a pathological diagnosis of central neurocytoma were included in the analysis. Extraventricular neurocytomas (previously known as cerebral neurocytomas) and pediatric patients were excluded. Demographics, clinical findings, pre- and post-operative radiological studies as well as histopathological reports, clinical follow-up with details of adjuvant treatments were analyzed. The study was approved by the institutional review board (ATADEK-2022/07).

### Pathology

Archived formalin-fixed paraffin-embedded pathology specimens of all patients were reviewed by one single neuropathologist (AED) according to the WHO 2021 criteria (2). Patients were diagnosed as “central neurocytoma (CN)” or “atypical central neurocytoma (aCN)” based on presence of atypical features and/or high Ki-67 index (>3%). Immunostaining for GFAP, OLIG-2, Synaptophysin, Neu-N, Ki-67 and p75 neurotrophin receptor (p75NTR) were performed for all specimens. Copy number status for MYCN was also studied in all specimens (FISH using ZytoLight ® SPEC MYCN/2q11 Dual Color Probe).

### Treatment and follow-up

All tumors were resected surgically. Either gross total resection (GTR) or subtotal resection (STR) was performed. There were no biopsies. The anterior interhemispheric transcallosal approach was used in all. For the single case of leptomeningeal dissemination, a pterional approach was used for removal of the tumor.

Gamma Knife radiosurgery and fractionated radiotherapy (RT) were utilized as adjuvant radiation treatments either at the time of recurrence/regrowth or upfront (if the pathology was reported as aCN with distinguishably high Ki-67 index). Prescribed stereotactic radiosurgery doses were 12 Gy and 14 Gy, both to the 50% isodose line. In fractionated radiotherapies, the total dose administered ranged between 40 and 56 Gy.

Patients underwent routine follow-up with MRI at first month and then every 6 months for 3 years and every year or until a new clinical symptom is encountered.

Regrowth, as defined, refers to the relapse of a tumor that underwent subtotal resection. Recurrence, on the other hand, is defined as the reappearance of a tumor in a local or distal location that gross total resection had been performed previously.

### Statistical analysis

SPSS version 25 (IBM, Armonk, New York, USA) was used for all statistical analyses. The chi-square, Kruskal–Wallis, Mann–Whitney U, log-rank with Benjamini-Hochberg method, Mantel-Haenszel test and *t*-tests were used to compare groups. Survival analyses were done with Kaplan–Meier plotter. The results were given with a 95% confidence interval. Statistical significance was set at a *p*-value of <0.05 for all analyses. Age, gender, tumor location and subtypes of the patients were used as variables for comparisons. Data visualization was carried out using RStudio (RStudio, PBC, Boston, MA, ABD).

## Results

### Clinical characteristics

The cohort consists of 33 adult patients. The median age was 30 years (min-max: 15–58 years) and the mean age was 30 ± 9 years. The mean age of CN was 30 ± 7 years and the mean age of aCN was 31 ± 12 years. The difference was not statistically significant (*p* = 0.772).

Nineteen patients were female and 14 were male (F/M ratio = 1.4). There were 13 females and 9 males (F/M ratio = 1.4) with typical pathological features. 6 females and 5 males (F/M ratio = 1.2) were classified as aCN.

The most common symptoms were headache (n = 29, 85%), dizziness (n = 6; 88%) and diplopia due to abducens nerve paralysis (*n* = 5; 15%). Clinical details are presented in Table 1.

**Table 1:** The demographic informations and clinical characteristics of the patients are presented.

Diagnosis	Age	Gender	First treatment	Adjuvant treatment	Ki-67 (%)	p75NTR	Localization	Recurrence/regrowth	Second treatment	Complications	PFS (months)	OS (months)
CN	32	F	GTR	No	2	Negative	RLV	No	No	No	227	227
CN	41	F	GTR	No	2	Negative	RLV	No	No	No	186	186
CN	34	M	GTR	No	4	Negative	LLV	No	No	No	180	180
CN	32	F	GTR	No	2	Negative	RLV	No	No	No	179	179
CN	34	M	GTR	No	1	Negative	LLV	No	No	No	177	177
CN	43	F	GTR	No	2	Negative	LLV	No	No	No	171	171
CN	29	F	GTR	No	1	Negative	RLV	No	No	No	131	131
CN	24	F	GTR	No	2	Negative	LLV	No	No	No	113	113
CN	22	F	GTR	No	N/A	Negative	RLV	No	No	No	111	111
CN	23	F	GTR	No	1	Negative	LLV	No	No	No	107	107
CN	34	M	GTR	No	2	Negative	RLV	No	No	No	87	87
CN	22	F	GTR	No	4	Negative	RLV	No	No	Hematom Hydrocephalus	72	72
CN	20	F	GTR	No	1	Negative	LLV	No	No	No	64	64
CN	21	M	GTR	No	1	Negative	Triventricular	No	No	No	63	63
CN	24	F	GTR	No	1	Negative	RLV	Local recurrence	RT (54Gy/30fr)	No	21	60
CN	37	M	GTR	No	1	Negative	RLV	No	No	No	60	60
CN	35	M	GTR	No	1	Negative	Third ventricle	No	No	No	60	60
CN	40	M	GTR	No	1	Negative	Triventricular	No	No	No	58	58
CN	32	M	GTR	No	6	Negative	RLV	No	No	No	46	46
CN	21	F	GTR	No	N/A	Negative	RLV	No	No	No	31	31
CN	36	F	STR	No	2	Negative	LLV	Regrowth	GKRS (14Gy to 50% il)	No	31	106
CN	24	M	STR	No	1	Negative	RLV	Regrowth	GKRS (12Gy to 50% il)	Hematoma Hydrocephalus	31	52
aCN	30	M	GTR	No	3	Negative	LLV	Local recurrence	GKRS (13Gy to 50% il)	No	51	164
aCN	23	F	GTR	No	1	Negative	LLV			No	101	101
aCN	47	F	GTR	No	8	p75NTR+	1. RLV 2. Sphenoid Wing	1. Local recurrence 2. Distal leptomeningeal recurrence	1. RT (56Gy/30fr) 2. GTR + RT (40Gy/15fr)	Hematoma	25	91
aCN	58	F	GTR	No	1	Negative	Third ventricle	No	No	No	84	84
aCN	26	F	GTR	No	6	Negative	LLV	No	No	No	82	82
aCN	23	M	GTR	No	8	Negative	LLV	No	No	No	63	63
aCN	20	F	GTR	No	4	Negative	Bilateral	No	No	No	62	62
aCN	26	M	GTR	No	2	Negative	Third ventricle	No	No	No	54	54
aCN	40	M	GTR	No	6	p75NTR+	LLV	No	No	No	4	4
aCN	18	M	GTR	RT (56Gy/30fr)	30	p75NTR+	RLV	No	No	No	15	15
aCN	26	F	STR	No	8	Negative	LLV	Regrowth	STR + RT (56Gy/30fr)	No	63	72

CN, central neurocytoma; aCN, atypical central neurocytoma; GTR, gross total resection; STR, subtotal resection; RT, radiotherapy; GKRS, Gamma Knife radiosurgery; OS, overall survival; PFS, progression-free survival; N/A, not available; RLV, right lateral ventricle; LLV, left lateral ventricle; M, male; F, female; Gy, gray; IL, isodose line; Fr, fraction.

**Figure 1:**
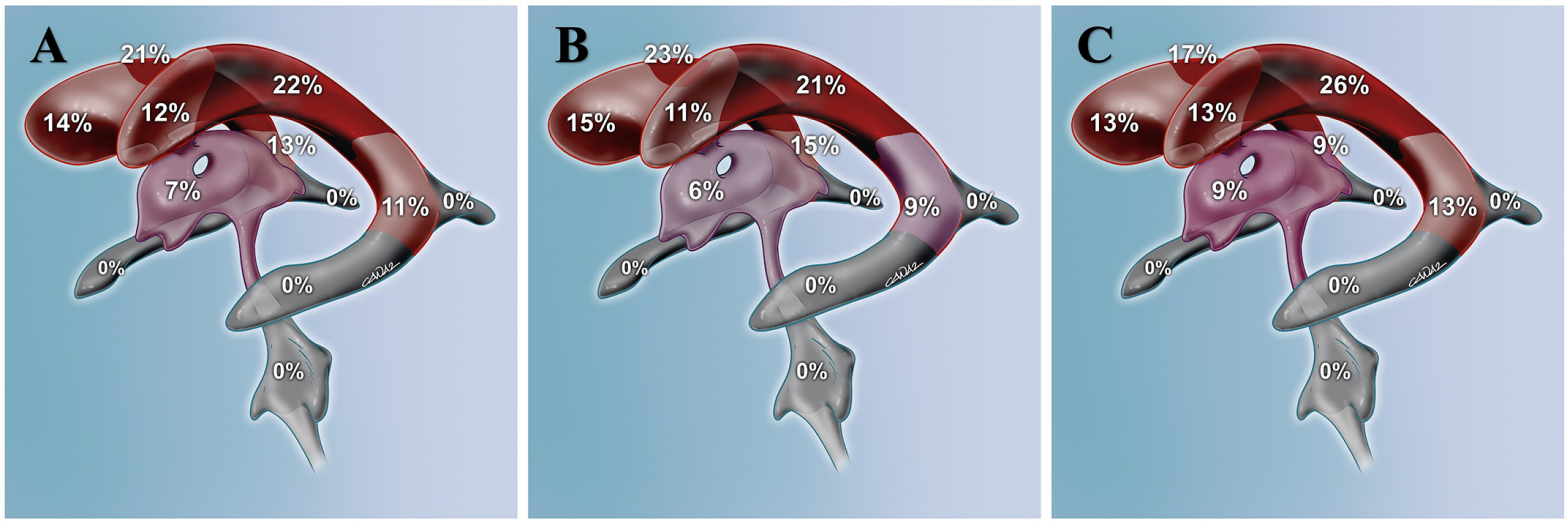
The distribution of central neurocytomas in the ventricular system, including the frontal horn, body, atrium, occipital horn, temporal horn, third, and fourth ventricles, is illustrated for all central neurocytomas in **(A)**, for typical central neurocytomas in **(B)**, and for atypical central neurocytomas in **(C)**.

Isolated right lateral ventricle involvement was observed in 14 (42%) patients, while left ventricular involvement was observed in 13 (39%) patients. One patient (3%) had bilateral ventricular involvement. Isolated third ventricular involvement was observed in 3 (9%) patients. Triventricular involvement was seen in 2 (6%) patients. The difference between localization of the typical and atypical histological groups was not statistically significant (*p* = 0.9641). Distribution of tumors along the ventricular system is presented in Table 1 and Figure 1.

### Pathological characteristics

Among the primary presentations, 67% of the cohort consisted of typical central neurocytomas (CN), while atypical histopathological features (aCN) were present in 33% of cases (11 of 33). Furthermore, there was no upgrading or malignant transformation observed in regrown or recurrent tumors.

The median Ki-67 labeling index for the entire group, CN cases and aCN cases were 6% (1–30%), 2% (1–6%), and 6% (1–30%), respectively. The mean Ki-67 values were 4±5%, 2±1%, and 7±8%, respectively. The Ki-67 index difference between CN and aCN cases was statistically significant (*p* = 0.0093). In cases which recurrence were seen (*n* = 3), the median Ki-67 index was found to be 4% (1–8%), with a mean Ki-67 of 3 ± 4%. In cases that showed regrowth (*n* = 3), the median of Ki-67 expression was 4% (1–8%), with a mean Ki-67 of 2 ± 4%, which were comparable to those observed in the recurrent group. The difference was not statistically significant (*p* = 0.774).

All cases, including both CN and aCN, showed positive immunostaining for Synaptophysin and Neu-n, indicating neuronal origin. On the other hand, immunostaining for GFAP and Olig-2 was negative.

Among aCN cases, including the metastatic case, three out of eleven cases (27%) demonstrated strong immunopositivity for p75NTR. In contrast, none of the typical CN cases showed p75NTR positivity.

### Treatment

A total of 40 surgeries were performed in 33 patients, which consisted of 35 tumor resections, 3 hematoma evacuations, and 2 ventriculoperitoneal shunting procedures. At the initial surgery, GTR was achieved in 30 of 33 cases, the rest (3 of 33, 9%) were STR. At the time of recurrence/regrowth two additional surgeries (one with GTR and the other with STR) were carried out. Three (3 of 35; 9%) surgeries were complicated by hematoma in the surgical bed. Two of these three patients (6% of the whole cohort) developed hydrocephalus and underwent additional ventriculoperitoneal shunting procedure. There was no surgical mortality (Table 1).

Gamma Knife radiosurgery was employed in 3 patients for post-regrowth following STR (*n* = 2, both CN) and post-local recurrence following GTR (*n* = 1, aCN). Fractionated radiotherapy was utilized to treat post-regrowth (*n* = 1, aCN), and post-recurrence (*n* = 1, CN). In another patient with aCN, Radiotherapy was used to treat local recurrence following GTR. Additionally, the same patient received RT again for distal leptomeningeal recurrence following the GTR of the lesion. Lastly, one patient with aCN received fractionated radiotherapy upfront due to both p75NTR positivity and high Ki-67 index (30%).

### Follow-up

**Figure 2:**
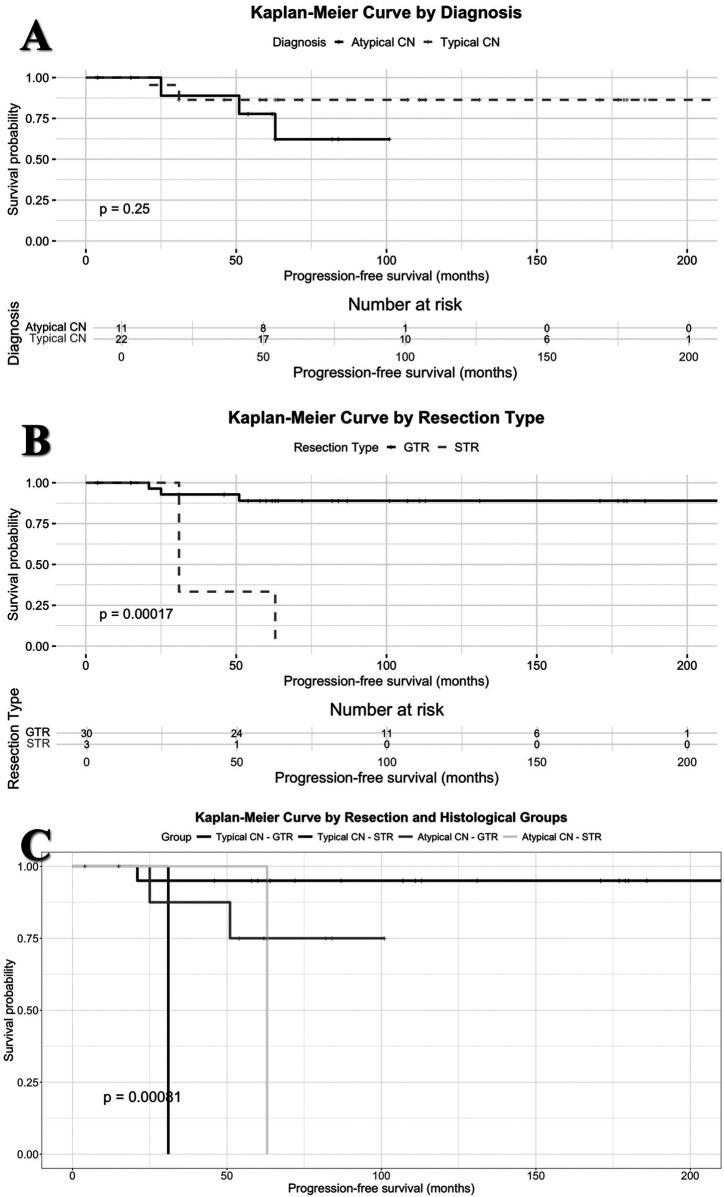
**(A)** Kaplan–Meier survival analysis comparing typical central neurocytoma and atypical central neurocytoma subtypes. **(B)** Kaplan–Meier survival analysis according to extent of resection (GTR vs. STR). **(C)** Kaplan–Meier survival analysis based on combined surgical extent and histopathological subtypes.

The mean follow up was 95 ± 54 months and median follow-up was 82 months (range: 4–227). 5-year disease-free survival rates for CN and aCN were 85% and 78%, respectively. 10-year disease-free survival rates were 67% for both. Kaplan–Meier survival analysis for these two histopathological subtypes is illustrated in Figure 2. The difference in progression-free survival between typical CN and atypical CN was not statistically significant (*p* = 0.25).

Regrowth was observed in all the three cases with STR. The median time to regrowth was 31 months (range: 31–63). One (33%) was aCN with a Ki-67 index of 8%. This particular tumor showed regrowth after 63 months, and the patient subsequently underwent additional STR and adjuvant radiotherapy administration. The remaining two tumors (67%) exhibited CN morphology. The time to regrowth for these two tumors was 31 months. Gamma Knife radiosurgery was used in the management of these two. Neither of the three tumors have shown signs of recurrence at the most recent follow-up. The follow-up times from the time of second treatment were 9 months, 21 months, and 75 months.

Recurrence was observed in 3 out of 30 primary tumors that underwent GTR, with a median time to recurrence of 25 months (range: 21–51). One (33%) of these tumors was CN and two (67%) were aCN. Recurrence was observed at 21 months in the CN case, and at 25 and 51 in the aCN cases. All recurrences received adjuvant radiation treatment. CN case received radiotherapy, one of the aCN cases was treated with Gamma Knife radiosurgery, and the remaining aCN received fractionated radiotherapy.

Kaplan–Meier survival analysis for resection groups is illustrated in Figure 2. The difference in progression-free survival between GTR and STR groups was statistically significant (*p* = 0.00017).

**Figure 3:**
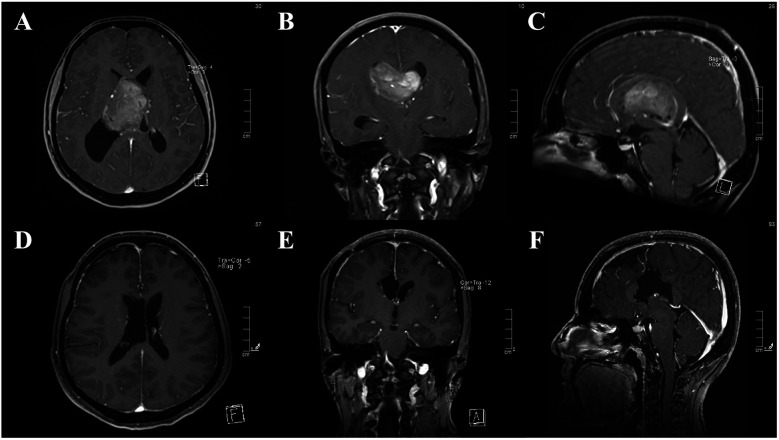
**(A–C)** Pre-operative contrast-enhanced MRI images of central neurocytoma located in both lateral ventricles. **(D–F)** Post-operative contrast-enhanced MRI images of gross total resection.

**Figure 4:**
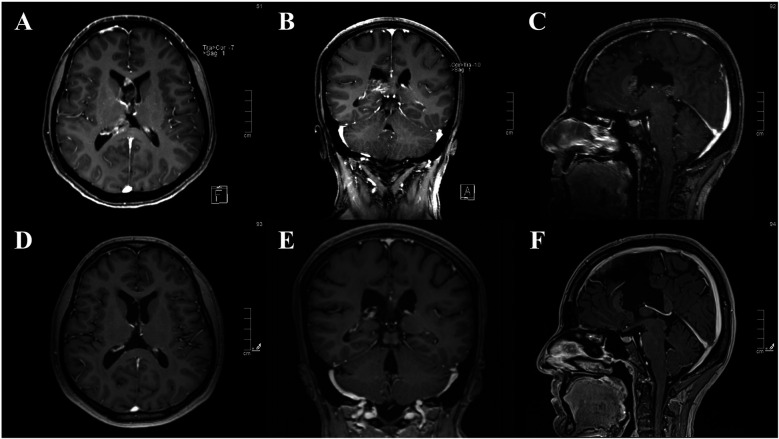
**(A–C)** contrast-enhanced MRI images of local recurrent central neurocytoma in the right lateral ventricle. **(D–F)** Post-RT contrast-enhanced MRI images of complete regression.

**Figure 5:**
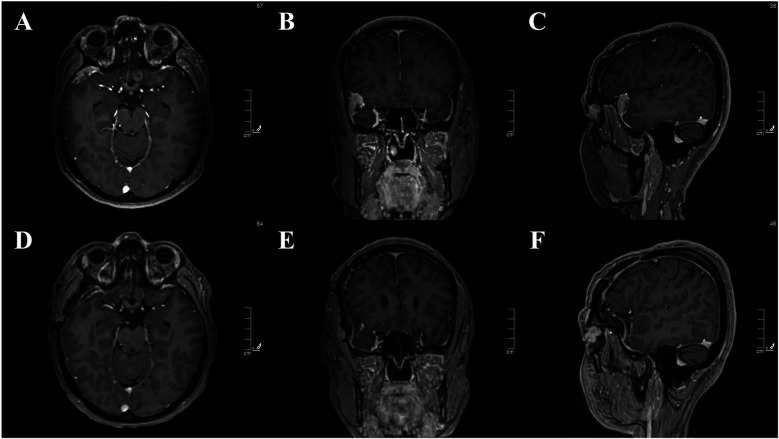
**(A–C)** Pre-operative contrast-enhanced MRI images of a solitary leptomeningeal dissemination of an previously gross total resected and irradiated aCN in the right sylvian fissure, atteched to the lateral sphenoid wing. **(D–F)** Post-operative contrast-enhanced MRI images of gross total resection.

Distal leptomeningeal dissemination was observed in 1 of 33 cases (3%). The primary tumor was aCN in the right lateral ventricle and treated with GTR (Figure 3). Adjuvant radiotherapy was applied after local recurrence in the right lateral ventricle. Despite the complete regression of the tumor (Figure 4), leptomeningeal dissemination in the right Sylvian cistern was observed after 48 months (Figure 5). It was subsequently treated with another GTR and adjuvant fractionated radiotherapy once again.

From pathological subtype perspective, among the 11 patients with aCN, one patient (9%) experienced regrowth after STR, and two patients (18%) showed recurrence after GTR. The mean time to regrowth/recurrence was 46 ± 19 months. Similarly, among the 22 patients with CN, two patients (9%) showed regrowth after STR, and one patient (5%) showed recurrence after GTR. The mean time to regrowth/recurrence was 28 ± 6 months.

When stratified into four groups based on both histopathological subtype and extent of resection (CN-GTR, CN-STR, aCN-GTR, aCN-STR), Kaplan–Meier survival analysis revealed a statistically significant difference in progression-free survival (*p* = 0.00081). Pairwise comparisons revealed that progression-free survival differed significantly between CN-GTR and CN-STR (*p* = 0.0032) as well as between CN-GTR and aCN-STR (*p* = 0.0208). No statistically significant difference was observed among other group combinations. Details pertaining to patients who experienced recurrence/regrowth are summarized in Table 1. Kaplan–Meier survival analyses are depicted in Figure 2.

## Discussion

### Markers of a more aggressive clinical course

Central neurocytomas are very rare tumors, hence limited information is available regarding their clinical course (1). They are generally considered to be benign tumors with a tendency for local invasion. However, aggressive courses including early recurrence, recurrence after GTR, and leptomeningeal dissemination can be observed, and it is generally associated with atypical central neurocytomas. The clinical determinants of such marked interpersonal variation in clinical course are not known and we sought to analyze several of those (10–14).

### Histopathological subtype

The meta-analysis conducted by Rades et al. demonstrated that aCN has a worse prognosis compared to CN. The 5-year OS and PFS rates were reported as 95% and 81%, respectively for CN cases, while they were 70% and 46% for aCN (23). In another study, CN had a 5-year PFS survival rate of 92%, whereas aCN had 65% (24). Interestingly, in the same study, two CN cases (13%) showed recurrence, and both recurrences occurred after GTR (24). However, in our series, the 5-year PFS rates were 85% and 78% for CN and aCN, respectively. 10-year PFS rates were 67% for both.

As per current understanding, an increased proliferative index (Ki-67 index) is considered to be the most important determinant of aCN, among other cellular atypia features including infiltrative tumor borders, pleomorphic structure, vascular proliferation, and necrosis (2). Söylemezoglu et al. reported 22% recurrence in cases with MIB-1 ≤ 2% and 63% in cases with MIB-1 > 2% during a 150 month follow-up (25). In our cohort, rates were 15% in Ki−67 ≤ 2% and 23% in Ki-67 > 2%. The difference was not statistically significant (*p* = 0.56). Also, despite the noticeably higher proportion of aCN in our cohort compared to the previously reported series, recurrence rates, regrowth rates, and progression intervals of both typical and atypical subtypes were similar (6, 15, 16, 23–43) (Table 2). Moreover, there was no statistical difference between the Ki-67 of those that recurred and those that did not.

**Table 2:** Previously reported series are presented in detail.

Publication	*N*	GTR (%)	Atypical cases (%)	Adjuvant irradiation (*N*)	Recurrences (%)	Follow-up (months)	Survival
Yasargil et al. (27)	8	88%	25%	3 (1 after recurrence)	38%	5–143 months	5-year PFS: 75%
Fujimaki et al. (28)	10	0%	N/A	10	0%	8–160 months	OS: 100%
Chen et al. (34)	10	60%	0%	6	0%	20 months (median)	No recurrence
Kim et al. (29)	15	47%	N/A	8 (2 with GTR, 5 with STR and 1 after recurrence)	13%	18–168 months	5-year PFS: 75%
Mackenzie et al. (31)	15	53%	20%	5 (2 after recurrence)	27%	13–255 months	5-year PFS: 43%
Mozes et al. (38)	17	18%	100%	12	82%	7–72 months	PFS: 2–36 months
Hallock et al. (33)	19	53%	37%	4	32%	1–262 months	10-year OS: 81% 10-year PFS: 57%
Figarella-Branger et al. (26)	20	45%	N/A	13	N/A	6–108 months	N/A
Sharma et al. (32)	20	70%	10%	15	0%	12–72 months	5-year OS: 44% 5-year PFS: 100%
Mattar et al. (41)	22	36%	100%	13 (11 upfront with STR)	36%	10–182 months	5-year OS: 35%
Alqroom et al.. (24)	30	77%	18%	6 (upfront in atypical cases)	3%	34–168 months	N/A
Schild et al. (30)	32	31%	N/A	16 (13 upfront and 3 after recurrence)	9%	2–180 months	5-year PFS for GTR: 100% 5-year PFS for STR: 70%
Samhouri et al. (42)	33	27%	N/A	19	21%	56 months (median)	5-year OS: 90% 5-year PFS: 76%
Soylemezoglu et al. (25)	36	94%	N/A	11 (2 primary treatment of choice)	22%	0–204 months	OS: 90%
Byun et al. (39)	40	63%	48%	10 (10 with STR)	20%	9–113 months	5-year PFS for typical CN: 92% 5-year PFS for aCN: 65%
Leenstra et al. (6)	45	47%	24%	14 (9 after recurrence)	33%	120 months (median)	10-year PFS for typical CN: 74% 10-year PFS for aCN: 46%
Kim et al. (36)	58	41%	N/A	14	N/A	18–304 months	5-year OS: 91% 10-year OS: 88%
Chen et al. (37)	63	38%	N/A	63	5%	15–129 months	5-year PFS for GTR + RT: 95% 5-year PFS for STR + RT: 96%
Wang et al. (15)	63	54%	N/A	14 (2 with GTR and 12 with STR)	16%	6–205 months	5-year PFS: 73% 10-year PFS: 58%
Han et al. (40)	67	82%	76%	33 (24 with GTR and 9 with STR)	6%	1–135 months	PFS time after GTR: 120–132 months PFS time after STR: 57–89 months
Vasiljevic et al. (35)	71	72%	18%	7 (2 with GTR)	13%	6–204 months	N/A
Xie et al. (16)	101	81%	64%	39 (38 RT and 1 GKRS)	N/A	0–132 months	5-year PFS for GTR: 92% 5-year PFS for STR: 60%
Zhang et al. (43)	413	42%	N/A	65	N/A	38–128 months	OS: 38–128 months
Rades et al. (23)	438	45%	20%	177 (43 with GTR and 134 with STR)	N/A	12–456 months	5-year PFS for typical CN: 81% 5-year PFS for aCN: 46%

CN, central neurocytoma; aCN, atypical central neurocytoma; *N* number of cases; GTR, gross total resection; STR, subtotal resection; RT, radiotherapy; OS, overall survival; PFS, progression-free survival; N/A, not available; *N*, number.

### The impact of the extent of resection and radiotherapy

STR is one of the markers for worse clinical course as all STR cases in this cohort, regardless of the typical or atypical variant, exhibited regrowth.

Almost all studies have reported that GTR is associated with improved outcomes compared to STR in terms of survival and rate of recurrence (15, 16, 23, 40). The 5-year OS and PFS rates in the GTR groups ranged between 92%–99% and 84%–92% respectively (16, 23). In contrast, the 5-year OS and PFS rates in the STR groups ranged between 72%–82% and 41%–60% respectively. In our series, the 5-year OS and PFS rates are 100% and 90% for GTR group, and 100% and 33% for the STR group. In the GTR cases, recurrence was significantly higher in aCN group with a rate of 18% compared to 5% of typical cases. Similarly, our results are consistent with the literature, with STR portending disease progression.

### Novel immunohistochemical markers of aggressive biology

p75NTR immunohistochemistry was conducted to investigate its potential correlation with aggressive behavior. Neurotrophins and their receptors, including p75NTR, play critical roles in regulating cell survival and proliferation in various cell types (44). P75NTR, also known as the low-affinity nerve growth factor receptor, belongs to the tumor necrosis factor (TNF) receptor family (45). Binding of nerve growth factor (NGF) to p75NTR can promote cell survival by activation of the nuclear factor kappa B (NF-*κ*B) transcription factor (44). Additionally, P75NTR has also been implicated as a mediator of invasion in human glioma cells, although its precise role remains unclear (46, 47). Forsyth et al. demonstrated that brain tumor-initiating cells (BTICs) express neurotrophin receptors (p75NTR, TrkA, TrkB, and TrkC) as well as their ligands (NGF, brain-derived neurotrophic factor, and neurotrophin 3) and secrete NGF (44). Downregulation of p75NTR has been shown to significantly decrease BTIC proliferation, while overexpression of p75NTR leads to increased proliferation (44). Similarly, in medulloblastoma, p75NTR expression has been associated with enhanced self-renewal, migration, and invasion of medulloblastoma cells in both *in vitro* and *in vivo* studies (47, 48).

In our study, over a quarter of the aCN, including the case with metastasis and the case with the highest mitotic index, exhibited strong immunoreactivity with p75NTR staining. While the overall number of positive cases was limited, it is noteworthy that one out of three p75NTR-positive cases (33%) experienced two recurrences. The median Ki-67 index in p75NTR-positive cases was 8% (range: 6%–30%), with a mean Ki-67 of 15% (±13 SD), which was higher compared to the overall cohort. Furthermore, the only case of leptomeningeal dissemination was observed in a p75NTR-positive case.

These findings suggest that p75NTR may serve as a potential marker for predicting aggressive clinical behavior in aCN. However, it is important to acknowledge that our study is limited by the small sample size, and the findings did not reach statistical significance. Therefore, these results should be interpreted with caution. Larger, prospective studies are warranted to confirm the role of p75NTR in the prognosis and biological behavior of central neurocytomas. Future investigations should also explore the underlying molecular mechanisms by which p75NTR may influence tumor aggressiveness.

## Conclusions

The clinical course of central neurocytoma ranges from typically indolent, locally invasive tumors to rare cases with leptomeningeal dissemination. Prognosis is influenced by multiple factors, bu our findings suggest that a high Ki-67 proliferative index alone does not necessarily predict aggressive tumor behavior. Gross total resection significantly improves outcomes; however, recurrence may still occur, even in histologically benign-appearing cases.

Histopathological atypia may help define the atypical variants (aCN), yet it does not reliably forecast clinical aggressiveness in long-term follow-up. Although our data indicate that p75NTR expression might be associated with more aggressive tumor biology, the limited number of cases precludes definitive conclusions. Thus, p75NTR cannot yet be regarded as a validated prognostic biomarker, and its potential role in CN progression warrants further investigation in larger, prospective cohorts.

Importantly, integrating conventional prognostic idicators -such as extent of resection and proliferative index- with emerging molecular markers like p75NTR may enhance risk stratification. This, in turn, supports the need for individualized follow-up strategies, particularly in patients with aCN, to better inform decisions regarding adjuvant therapies and improve long-term clinical outcomes.

## Limitations

This study has several limitations that should be acknowledged. First, the retrospective nature of the study introduces inherent selection bias and limits the ability to establish causal relationships. Additionally, although our cohort of 33 patients represents one of the largest single-institution series of adult central neurocytomas reported to date, the sample size remains relatively small, particularly for subgroup analyses. This limitation is particularly relevant for the assessment of p75NTR as a prognostic marker, as the number of positive cases was low, preventing robust statistical validation.

Another limitation is the heterogeneity in treatment modalities over the 25-year study period, which may have influenced outcomes. Advances in neurosurgical techniques, radiotherapy, and adjuvant treatments during this time may have contributed to variations in patient management and prognosis. Moreover, although we performed immunohistochemical analysis of p75NTR expression, we did not conduct molecular or genetic studies to further elucidate its potential role in tumor progression. Future studies incorporating genomic and transcriptomic profiling may provide deeper insights into the biological significance of p75NTR in central neurocytoma.

Lastly, the lack of long-term functional outcome data limits our ability to assess the impact of different treatment strategies on patients' quality of life and neurological function. Prospective, multicenter studies with larger cohorts and standardized treatment protocols are needed to confirm our findings and further explore the clinical significance of p75NTR in central neurocytomas.

## Data Availability

The original contributions presented in the study are included in the article/Supplementary Material, further inquiries can be directed to the corresponding author.
